# An Integrated Three-Long Non-coding RNA Signature Predicts Prognosis in Colorectal Cancer Patients

**DOI:** 10.3389/fonc.2019.01269

**Published:** 2019-11-22

**Authors:** Yuhang Liu, Bingxin Liu, Guoying Jin, Jia Zhang, Xue Wang, Yuyang Feng, Zehua Bian, Bojian Fei, Yuan Yin, Zhaohui Huang

**Affiliations:** ^1^Wuxi Cancer Institute, Affiliated Hospital of Jiangnan University, Wuxi, China; ^2^Laboratory of Cancer Epigenetics, Wuxi School of Medicine, Jiangnan University, Wuxi, China; ^3^Department of Surgical Oncology, Affiliated Hospital of Jiangnan University, Wuxi, China

**Keywords:** long non-coding RNA, overall survival, prognosis, biomarkers, training set, clinical validation set

## Abstract

Colorectal cancer (CRC) is one of the most common cancers worldwide, whose morbidity and mortality gradually increased. Here, we aimed to identify and access prognostic long non-coding RNAs (lncRNAs) associated with overall survival (OS) in CRC. Firstly, RNA expression profiles were obtained from The Cancer Genome Atlas (TCGA) database, and 439 CRC patients were enrolled as a training set. Univariate Cox analysis and the least absolute shrinkage and selection operator analysis (LASSO) were performed to identify the prognostic lncRNAs. Multivariable Cox regression analysis was used to establish a prognostic risk formula including three lncRNAs (AP003555.2, AP006284.1, and LINC01602). The low-risk group had a better OS than the high-risk group (*P* < 0.0001), and the areas under the receiver operating characteristic curve (AUCs) of 3- and 5-year OS were 0.712 and 0.674, respectively. Then, we evaluated the signature in a clinical validation set which were collected from the Affiliated Hospital of Jiangnan University. Compared with the low-risk group, patients' OS were found to be significantly worse in the high-risk group (*P* = 0.0057). The AUCs of 3- and 5-year OS were 0.701 and 0.694, respectively. Finally, we constructed an lncRNA–microRNA (miRNA)–messenger RNA (mRNA) competing endogenous RNA (ceRNA) network to explore the potential function of three differentially expressed lncRNAs (DElncRNAs). The Kyoto Encyclopedia of Genes and Genomes (KEGG) pathway analysis indicated that these DElncRNAs were involved with several cancer-related pathways. In summary, our data provide evidence that the three-lncRNA signature could serve as an independent biomarker to predict prognosis in CRC. This study will also suggest that these three lncRNAs potentially participate in the progression of CRC.

## Introduction

Colorectal cancer (CRC) is a worldwide public health problem with the fourth highest incidence and second highest mortality in malignant tumors (1). The occurrence and progression of CRC were associated with multiple and complex factors, including lifestyle (2, 3), living surroundings (4), and genetic and epigenetic alterations (4, 5). Although therapeutic methods for CRC have largely improved in recent years, prognosis remains unsatisfactory due to individual differences (6). Thus, it is necessary to find sensitive and precise biomarkers to better predict survival and prognosis of CRC patients.

Many studies showed that genes are no longer a piece of DNA that encodes a protein but also transcripts RNA that is not translated into protein (7–9). With the development of sequencing technology, abundant novel non-coding RNAs have been found (10). Long non-coding RNAs (lncRNAs), a kind of non-coding RNAs with length greater than 200 nucleotides (11, 12), were initially considered as the “noise” of genome transcription, a by-product of RNA polymerase II transcription, and had no biological function. Nevertheless, accumulating researches manifested that lncRNA expression or dysfunction was associated with a variety of major human diseases, including cancers (13–17). Rigoutsos et al. found that lncRNA N-BLR could promote cell migration and facilitate CRC invasion (18). Our previous study identified SNHG6 as an oncogene and predicted poor prognosis in CRC (19); we also found that the lncRNA LINC00152 could promote CRC cell proliferation and metastasis (20). Another study from our group suggested that lncRNA-FEZF1-AS1 promoted CRC cell proliferation and metastasis (21). Ozawa et al. identified lncRNAs CCAT1 and CCAT2, regarded as prognostic biomarkers in CRC (22). However, the biological function of lncRNAs in CRC is still not well-known, and a systematic study incorporating multiple lncRNAs' expression is still needed for improving the predictive accuracy of CRC prognosis.

Since a competing endogenous RNA (ceRNA) hypothesis, microRNAs (miRNAs) and lncRNAs could interact with each other through miRNA response elements (MREs), was proposed in 2011 (23), more studies have suggested that an lncRNA–miRNA–messenger RNA (mRNA) network associated with the pathomechanism of multiple cancers (24–29). LncRNAs could act as sponges or ceRNAs of miRNAs to regulate tumorigenesis and metastasis of CRC (30–37). Many studies have suggested that ceRNAs participate in prognosis of different cancers. What's more, studies on the mechanism of ceRNAs have potential clinical diagnostic and prognostic value. They could be potential prognostic biomarkers or novel therapeutic targets.

In this study, we identified three potential prognostic lncRNAs in CRC and confirmed the integrated three-lncRNA signature as an independent prognostic biomarker in the clinical validation set. Furthermore, we constructed an lncRNA–miRNA–mRNA network to investigate possible biological functions of lncRNAs in CRC.

## Materials and Methods

### Data Treating

The data of RNA expression profiles and clinical information for CRC were downloaded from The Cancer Genome Atlas (TCGA) database (https://portal.gdc.cancer.gov/repository), including 482 CRC tissues and 42 non-tumor tissues. LncRNAs were annotated by human gene annotation files (GRCh38.p12), which was downloaded from the Ensembl database (https://asia.ensembl.org/index.html). The expression data of lncRNAs were analyzed by the R/Bioconductor package edgeR. Differentially expressed lncRNAs (DElncRNAs) were distinguished according to a |log 2 fold change| >1.5 and a *P*-value < 0.05. Then hierarchical clustering analysis was performed by the R package pheatmap. A volcano plot was drawn using the R software.

### Characterization of lncRNAs Associated With Overall Survival

We excluded CRC patients from TCGA dataset according to the following criteria: (1) patients without information of survival status and survival time; (2) patients without complete lncRNA expression data. Finally, 439 CRC patients were selected in our study. Among these patients' tissues, 42 CRC tissues and their paired adjacent non-cancerous tissues were used to identify DElncRNAs. And all the 439 patients were grouped into the training set.

Univariate Cox analysis was performed to assess the association between the expression levels of DElncRNAs and the overall survival (OS) of patients, and lncRNAs with a *P*-value < 0.05 were selected for further analysis. Then the least absolute shrinkage and selection operator (LASSO) model was used to find vital lncRNAs from the prognostic DElncRNAs. The LASSO method was utilized by the package “glmnet” in the R (version 3.5.1) software. Then Kaplan–Meier analysis was performed to single out lncRNAs significantly associated with the OS from DElncRNAs, which were selected by the LASSO method.

### Establishment of Prognostic Risk Score Formula

In light of the expression level of lncRNAs and regression coefficients, a prognostic risk formula was established by multivariable Cox regression analysis. The random forest plot was performed using the R package survminer. The risk scores of each patient were calculated by the formula as mentioned above. Finally, all patients were divided into a high-risk group and a low-risk group by utilizing the median risk score as the cutoff value.

### Assessing the Prognostic Risk Score Model

Next, a Kaplan–Meier survival curve was used to evaluate the prognosis between the low-risk group and high-risk group. And a time-dependent receiver operating characteristic (ROC) curve was performed to assess the diagnostic accuracy based on the risk score for 3- and 5-year OS probability. A *P*-value < 0.05 was recognized as statistically significant. A concordance index (C-index) was calculated to estimate the value of prognostic risk formula by the R package survcomp. The expression profiles of key lncRNAs in the high-risk and low-risk groups were plotted by risk heatmap. The above analyses were employed using R (version 3.5.1).

### Clinical Samples and Quantitative RT-PCR Assay

In the clinical validation set, we chose CRC patients based on the following criteria: (1) patients treated in the Affiliated Hospital of Jiangnan University; (2) patients with complete follow-up information, including survival status and survival time; (3) patients who did not receive treatment before surgery. Eighty-five CRC patients were gained from the Affiliated Hospital of Jiangnan University as the clinical validation set. The clinical information of all patients was listed in Table 1. All patients signed the informed consent about using their tumor tissues, and this study was ratified by the clinical research ethics committees of the participating institutions.

**Table 1:** Clinicopathological characteristics of 85 patients with colorectal cancer (CRC) in the validation set.

**Characteristics**	**CRC patients (*****N*** **=** **85)**	
	***n***	**%**
**Age (years)**		
≥60	47	55.29
<60	38	44.71
**Gender**		
Male	47	55.29
Female	38	44.71
**AJCC stage**		
I	23	27.05
II	36	42.35
III	26	30.60
IV	0	
**AJCC-T**		
T1	4	4.70
T2	22	25.88
T3	39	45.88
T4	20	23.54
**AJCC-N**		
N0	59	69.41
N1	20	23.53
N2	6	7.06
N3	0	0
**AJCC-M**		
M0	84	98.82
M1	1	1.18

Total RNA of tumor tissues was isolated using RNAiso Plus (Takara, Japan). NanoDrop 2000 (Thermo, USA) was used to measure RNA concentrations. Total RNA was reverse transcribed to complementary DNA (cDNA) using the Prime-Script II 1st Strand Synthesis Kit (TaKaRa). The expression levels of lncRNAs were quantitated using the UltraSYBR Mixture (CWBIO, China) by the ViiA7 real-time PCR system. The lncRNA expression level was calculated as follows: ΔCT = CT (lncRNA) – CT (β-actin). The sequences of used primers are listed in Supplementary Table 1.

### Construction of the CeRNA Network

The miRNAs which potentially interacted with three DElncRNAs were predicted using LncBOOK (http://bigd.big.ac.cn/lncbook), a curated information database of human lncRNAs. Finally, a total of 1,039 miRNAs were identified. Next, according to the score of interaction between lncRNAs and miRNAs, we selected the top 30 closely related miRNAs of each lncRNAs for further study. Then the target mRNAs of miRNA were predicted by utilizing miRDB (http://www.mirdb.org/), TargetScan (http://www.targetscan.org/), and miRTarBase (http://mirtarbase.mbc.nctu.edu.tw/php/index.php). Finally, we established the lncRNA–miRNA–mRNA network by using the Cytoscape software (version 3.6.1).

### Functional Prediction

The Kyoto Encyclopedia of Genes and Genomes (KEGG) pathway analysis was performed to describe the potential function of differentially expressed mRNAs (DEmRNAs) by the package “clusterProfiler” (http://bioconductor.org/packages/clusterProfiler/) in the R (version 3.5.1) software.

### Statistical Analysis

Both the expression profiles of RNAs and clinical information data were excavated from TCGA database by the Perl (version 5.24.3) software and R (version 3.5.1) software. All statistical analyses were carried out using SPSS23.0 (SPSS, Chicago, IL, United States) or R software. A *P*-value < 0.05 was regarded as statistically significant.

## Results

### DElncRNAs in CRC

**Figure 1:**
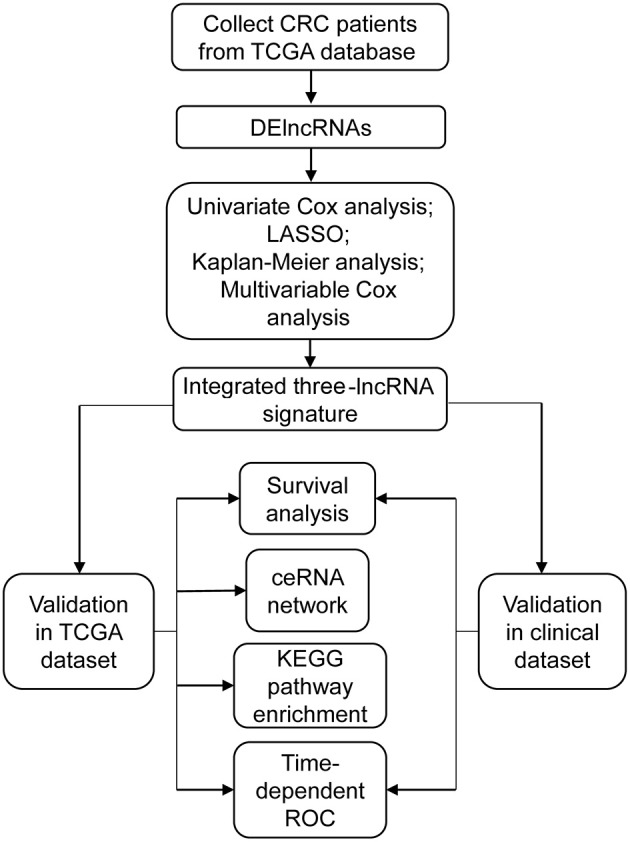
The main flowchart of this study. CRC, colorectal cancer; DElncRNAs, differentially expressed long non-coding RNAs; LASSO, least absolute shrinkage and selection operator analysis.

**Figure 2:**
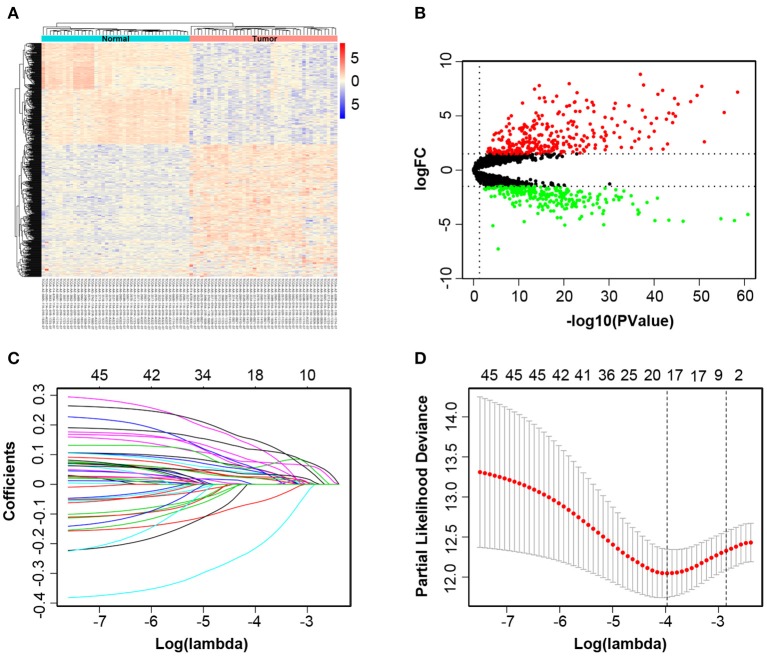
Identification of different expressions of lncRNAs associated with colorectal cancer (CRC). The heatmap and volcano plot of differentially expressed long non-coding RNAs (DElncRNAs) between 42 CRC tissues and their paired adjacent non-cancerous tissues **(A,B)**. Least absolute shrinkage and selection operator analysis (LASSO) coefficient profiles of 46 DElncRNAs selected by univariate Cox regression analysis **(C,D)**.

The overview of the screening strategy used in this study is displayed in Figure 1. In the 439 tissues of CRC patients, 42 CRC tissues and their paired adjacent non-cancerous tissues were utilized to screen DElncRNAs. Then 580 DElncRNAs, with a critical point of |logFC > 1.5| and *P*-value < 0.05, were gained by using the R/Bioconductor package edgeR. Among the DElncRNAs, 319 DElncRNAs are upregulated and 261 DElncRNAs are downregulated (Supplementary Table 2). The heatmap and volcano plot were shown respectively in Figures 2A,B.

### Recognition of Key lncRNAs Correlated With OS

**Figure 3:**
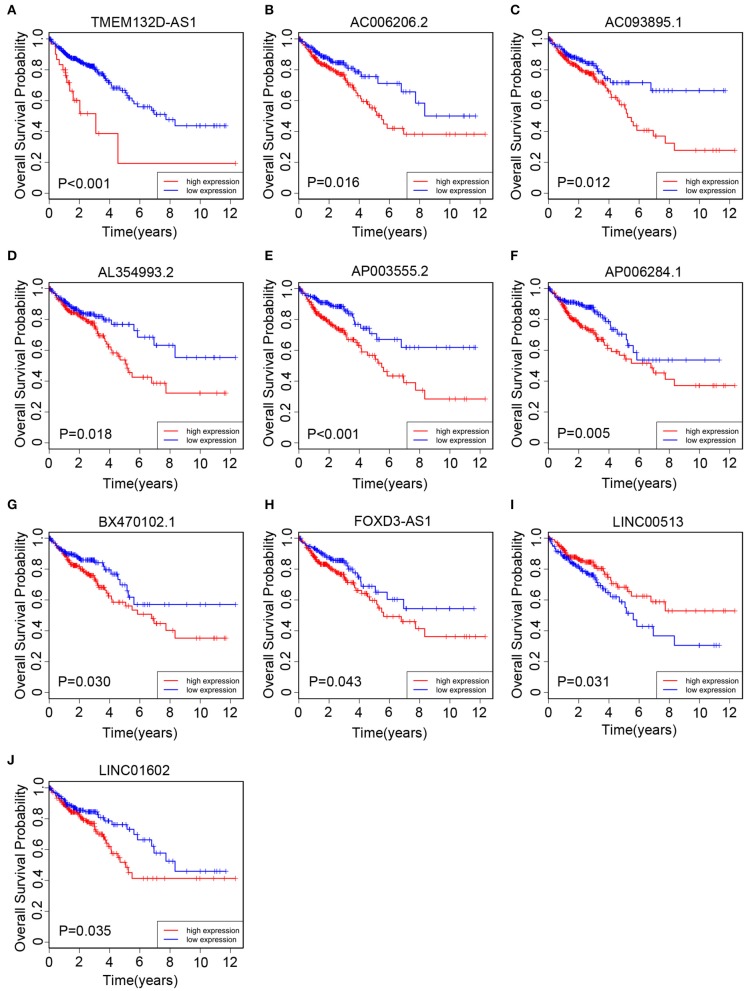
The Kaplan–Meier curve of 10 prognostic lncRNAs in CRC patients collected from The Cancer Genome Atlas (TCGA) cohort. The Kaplan–Meier curve for **(A)** TMEM132D-AS1, **(B)** AC006206.2, **(C)** AC093895.1, **(D)** AL354993.2, **(E)** AP003555.2, **(F)** AP006284.1, **(G)** BX470102.1, **(H)** FOXD3-AS1, **(I)** LINC00513, and **(J)** LINC01602.

**Figure 4:**
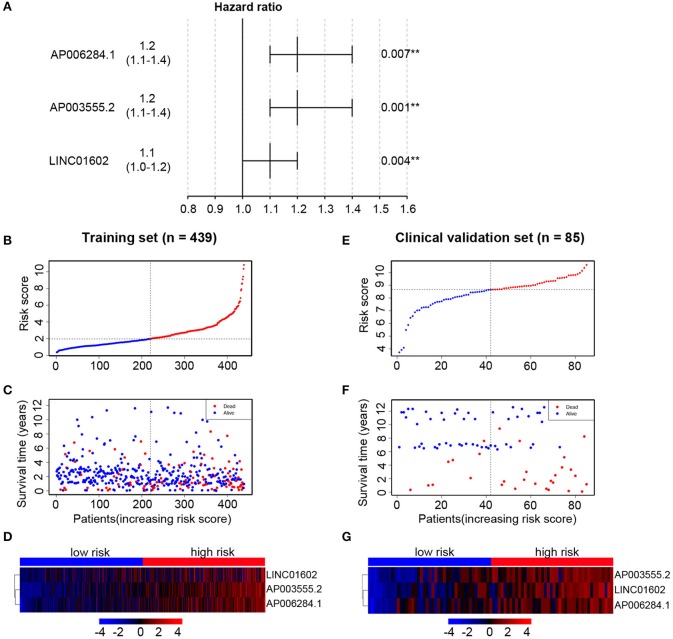
Multivariable Cox regression analysis is performed to select key long non-coding RNAs (lncRNAs) and the distribution of risk score, survival status, and risk heatmap of three prognostic lncRNAs in the training set and clinical validation set, respectively. **(A)** Hazard ratio of selected key lncRNAs. In the training set, the risk score distribution of three lncRNAs **(B)**; the overall survival status of 439 patients **(C)**; and expression heatmap of three lncRNAs in the low-risk and high-risk groups **(D)**. In the clinical validation set, the risk score distribution of three lncRNAs **(E)**; the overall survival status of 85 patients **(F)**; and expression heatmap of three lncRNAs in the low-risk and high-risk groups **(G)**.

All of 439 CRC patients collected from TCGA database were regarded as the training set. Next, we performed univariate Cox regression analysis to estimate the prognostic relationship between lncRNA expression profiles and patient OS. Among 580 DElncRNAs, 46 lncRNAs with a *P*-value < 0.05 were selected for the following study. Next, the LASSO method was performed, and the coefficients of 46 lncRNAs were shown in Figure 2C, and the minimize λ method screened out 17 lncRNAs (Figure 2D). We then used Kaplan-Meier survival curves to further analyze the relationship between the 17 lncRNAs and the OS of patients. Finally, we found that 10 lncRNAs were observably related with OS (Figure 3), and the other seven lncRNAs were not significant (Supplementary Figure 1). Using multivariable Cox regression analysis, three lncRNAs (AP003555.2, AP006284.1, and LINC01602) were identified as integrated prognostic biomarkers for CRC patients (Figure 4A). The results showed that all lncRNAs play as hazards with positive coefficients. The relative expression levels of the three lncRNAs between 42 CRC tissues and their paired adjacent non-cancerous tissues were displayed in Supplementary Figure 2.

### Establishment of Prognostic Risk Score Formula in the Training Set

We then established a prognostic risk score formula in the training set based on the expression profiles of three prognostic lncRNAs and their regression coefficients. The prognostic risk score formula was as follows: risk score = 0.2212 * (the expression level of AP003555.2) + 0.2081 * (the expression level of AP006284.1) + 0.1214 * (the expression level of LINC01602). The risk scores were calculated for all patients and split patients into a high-risk group (*n* = 219) and a low-risk group (*n* = 220) by using the median risk score as the cutoff value. The distribution of risk scores and survival status of the patients were shown in Figures 4B,C. The expression profiles of three lncRNAs were shown using a risk heatmap in 439 ordered patients (Figure 4D). These results suggested that patients with higher risk scores currently had worse OS than those with lower risk scores.

### Prognostic Value of the Three lncRNAs Respectively in the Clinical Validation Set

**Figure 5:**
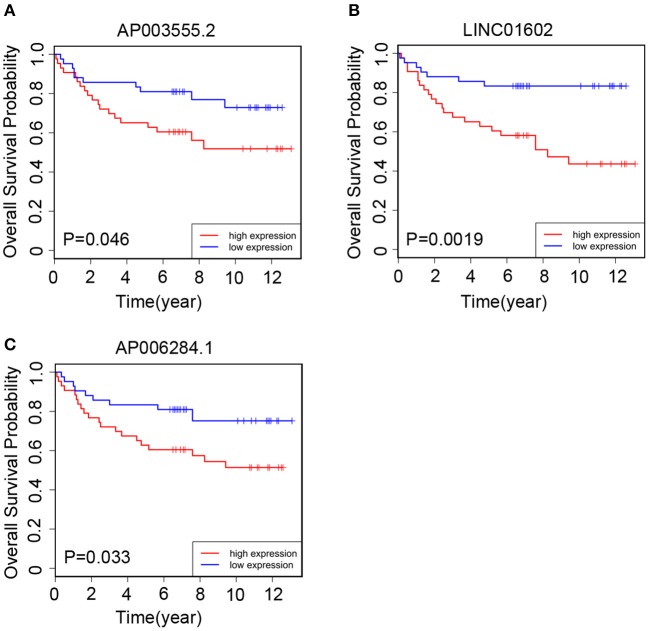
The Kaplan–Meier curve of three prognostic long non-coding RNAs (lncRNAs) respectively in the clinical validation set. In the validation set, the Kaplan–Meier curve for **(A)** AP003555.2, **(B)** LINC01602, and **(C)** AP006284.1.

We collected 85 CRC patients from the Affiliated Hospital of Jiangnan University as the clinical validation set. Next, we quantitated the relative expression levels of the three lncRNAs in each patient's cancer tissue by quantitative RT-PCR assay and used Kaplan-Meier analysis to evaluate the prognostic capacity. As a result, we found that the high-expression group had a worse OS than the low-expression group based on the median expression level as the cutoff value for each lncRNA (Figures 5A–C). The results were in accord with those of TCGA cohort (Figures 3E,F,J).

### Prognostic Value of the Three-lncRNA Signature in the Training and Clinical Validation Sets

**Figure 6:**
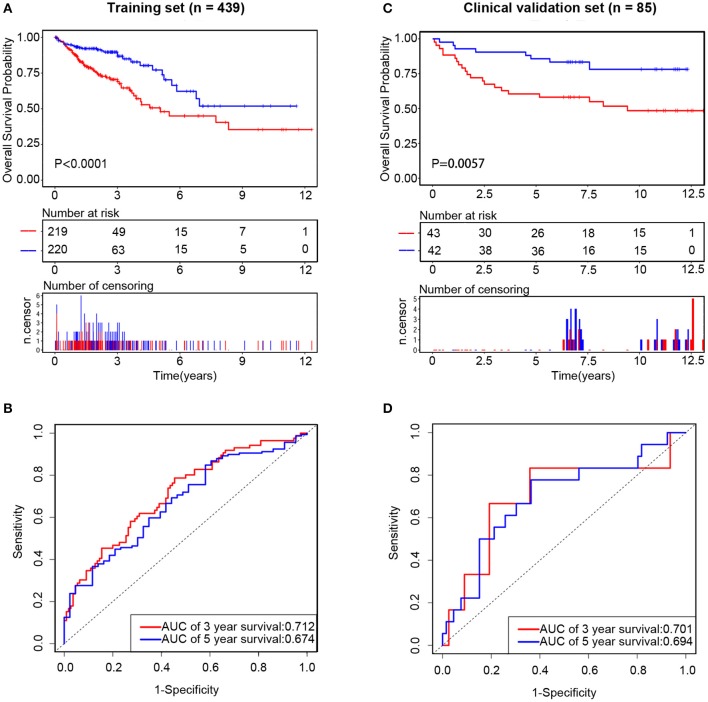
The prognostic value of integrated three long non-coding RNAs (lncRNAs) in the training set and clinical validation set, respectively. In the training set, the Kaplan–Meier curve of the overall survival (OS) between the low-risk and high-risk groups split by median risk score **(A)** and time-dependent receiver operating characteristic (ROC) analysis for the 3- and 5-year OS probability **(B)**. In the clinical validation set, the Kaplan–Meier curve of the OS between the low-risk and high-risk groups split by median risk score **(C)** and time-dependent ROC analysis for the 3- and 5-year OS probability **(D)**.

Subsequently, we assessed the prognostic value of the above risk formula in the training set by using Kaplan-Meier analysis. We found that the low-risk group had a better OS than the high-risk group (*P* < 0.0001) (Figure 6A). Moreover, time-dependent ROC analysis was also utilized to evaluate the prognostic capacity of the risk formula. The areas under the ROC curve at 3 and 5 years were 0.712 and 0.674, respectively, which suggested that the integrated three-lncRNA signature had better utility than each single one (Figure 6B). The value of the C-index was 0.65 (95% CI: 0.59–0.72), showing a fine prognostic value on predicting patients' survival.

In order to confirm the common prognostic value of the integrated three-lncRNA signature in different patient cohorts, our risk model was assessed in the clinical validation set. Eighty-five patients were divided into a high-risk group (*n* = 43) and a low-risk group (*n* = 42) by using the above three-lncRNA signature.

Compared with that in the low-risk group, patients' OS was found to be significantly worse in the high-risk group. The risk scores were calculated, and the distribution were shown in Figures 4E,F. A risk heatmap in 85 ordered patients was shown in Figure 4G. As expected, Kaplan-Meier analysis suggested that the low-risk group had a better OS than the high-risk group (*P* = 0.0057) (Figure 6C). Time-dependent ROC analysis indicated that the areas under the ROC curve at 3 and 5 years were 0.701 and 0.694, respectively (Figure 6D). These results were consistent with those of the training set.

### Function of Three lncRNAs

To explore the potential roles of the three lncRNAs in CRC, we constructed an interactional network of lncRNA–miRNA–mRNA. Firstly, utilizing the LncBOOK online analysis tool, we predicted the target relationship among three lncRNAs and miRNAs. We then chose the top 30 target miRNAs of each lncRNA, according to the score of interaction, to predict the downstream target mRNAs by using miRDB, TargetScan, and miRTarBase. In the end, we constructed a ceRNA network including 3 lncRNAs, 90 miRNAs, and 1,791 mRNAs (Supplementary Figure 3A).

Next, we employed the KEGG pathway analysis to explore the potential mechanisms involved in the progression of CRC using the package ClusterProfiler in the R software based on the selected mRNAs. The top 15 KEGG pathways were shown in Supplementary Figure 3B. Some key cancer-related pathways were found, such as the PI3K-Akt pathway, TGF-β pathway, and CRC and P53 pathways.

## Discussion

Currently, a growing number of studies have demonstrated that dysregulated lncRNAs are involved in the progression of CRC and might be potential biomarkers for diagnosis, treatment, and prognostic judgment of the disease. Compared with single biomarkers, a system of multiple integrated biomarkers can improve predictive accuracy. In our present study, a novel three-lncRNA signature was constructed for predicting OS of CRC patients. Using multiple appropriate statistical methods, three lncRNAs (AP003555.2, AP006284.1, and LINC01602) were identified as independent prognostic indicators. Then, an integrated three-lncRNA signature was constructed based on their expression profiles and regression coefficients. The value of prognosis was confirmed by two independent patients' cohort. In TCGA training set, patients were divided into low-risk and high-risk groups based on the prognostic score calculated by the above lncRNA signature. Survival curves indicated significant differences in the patients' OS between the two groups. Time-dependent ROC analysis indicated that the lncRNA signature had high predictive accuracy in predicting OS. A clinical validation set was used to verify the universal applicability of the model.

All the three lncRNAs were already annotated by the Ensembl database. However, little was known about their roles in tumorigenesis and progression. Zhang et al. found that a decreased expression of LINC01602 was associated with worse survival in patients with rectal adenocarcinoma (38). No public reports were found for the other two lncRNAs (AP003555.2 and AP006284.1) according to a PubMed search. All three lncRNAs were upregulated in CRC tissues compared with normal tissues. Meanwhile, a high expression of each DElncRNA was significantly associated with poor prognosis in CRC. Importantly, multivariable Cox regression analysis confirmed their prognostic role for CRC. Our results suggest that the three DElncRNAs may play crucial roles in the pathomechanism of CRC and act as potential prognostic biomarkers.

In contrast with previous studies, the integrated three-lncRNA signature reported here was distinctly different. First of all, we used 42 CRC tissues and their paired adjacent non-cancerous tissues selected from TCGA database as self-control to analyze DElncRNAs. In this way, it is effective to enhance the balance between groups, control confounders, and improve the accuracy of research. The most important point was that we collected CRC patients from the Affiliated Hospital of Jiangnan University as the clinical validation set to assess the three-lncRNA signature. Previous studies usually constructed a prognostic model as follows (39–43): (1) using one GEO database to build a prognostic model and using another GEO database or TCGA database as a validation set to evaluate the constructed model; (2) using TCGA database to establish a training model and using a GEO database to validate; and (3) dividing TCGA database randomly into two data sets equally or unequally, one as a training set and the other as a validation set. Generally, most studies were based on the online data from publicly available databases without validating an additional clinical patient cohort. In other words, there was a lack of evidence that the results of bioinformatic analysis, such as RNA-Seq, were well-validated in external patients' cohort by quantitative RT-PCR assay. In our study, we used TCGA data as the training set to establish an lncRNA model associated with OS. We then collected 85 CRC patients as the clinical validation set to evaluate whether the model was also appropriate in clinical samples. Interestingly, the results showed that the model constructed from TCGA dataset had a common prognostic value in the clinical patients' cohort.

CeRNA hypothesis revealed that lncRNAs could competitively bind miRNAs, as a “sponge” of miRNAs, and then indirectly regulate the downstream target genes of miRNAs (23). The lncRNA SNHG6 could sponge miR-26a/b and miR-214 to regulate EZH2 expression and thus promote CRC cell growth, migration, and invasion (30). An oncogenic lncRNA, NEAT1, was a “sponge” in CRC to competitively bind miR-193a-3p and thus upregulate the expression of the downstream gene *KRAS* (31). Liu et al. found that linc01296 functions as a sponge of miR-26a to regulate the expression of its target gene *MUC1* and regulate the activity of the PI3K/AKT signal pathway (35). In order to explore the potential biological function of the three lncRNAs, we constructed an lncRNA–miRNA–mRNA ceRNA network. We predicted the target miRNAs of each lncRNA and used the intersection of three databases (miRDB, TargetScan, and miRTarBase) to predict target mRNAs. KEGG pathway analysis indicated that the three lncRNAs' functions were potentially associated with several cancer-related pathways, such as the “PI3K-Akt signaling pathway,” “TGF-β signaling pathway,” “Colorectal cancer,” and “P53 signaling pathway.” Current studies have shown that the PI3K-Akt pathway is activated in many types of cancers. Many studies have indicated that the PI3K-Akt pathway plays a vital role in cell proliferation, migration, and invasion in CRC (35, 44, 45). Meanwhile, several studies have indicated that the TGF-β signaling pathway was also associated with lncRNAs; for example, the lncRNA SNHG6 could accelerate cell growth, invasion, and migration via activating the TGF-β signaling pathway in CRC (46). Here, we constructed an interactional network of lncRNA–miRNA–mRNA which may be involved in the progression of CRC, but the potential mechanisms still need to be investigated in future studies.

Although a three-lncRNA signature was constructed and appears to be a potential prognostic biomarker in clinical application, there are also some limitations. First, the sample size in our clinical validation set was not large enough. The second limitation was that the prognostic value of the three-lncRNA panel is not very satisfactory. Third, the binding affinities between lncRNAs and miRNAs, miRNAs, and mRNAs were predicted only by online databases and should be further experimentally investigated. Fourth, the biological functions of the three DElncRNAs were not assessed in this study.

In conclusion, we constructed an integrated three-lncRNA signature that was significantly associated with OS in CRC patients which could accurately identify patients with low prognostic risk from those with high prognostic risk. Furthermore, we evaluated the accuracy and reliability of the above signature in our own clinical validation set. These results suggested that the integrated three-lncRNA signature could potentially act as a prognostic model in CRC.

## Data Availability Statement

Publicly available datasets were analyzed in this study. This data can be found here: https://portal.gdc.cancer.gov.

## Ethics Statement

The studies involving human participants were reviewed and approved by Medical Ethics Committee of Affiliated Hospital of Jiangnan University. The patients/participants provided their written informed consent to participate in this study.

## Author Contributions

ZH, YY, and YL designed and conceived the study. YL and BL performed majority of analyses for the study. GJ, JZ, and XW downloaded the data from an online database. YF and ZB performed the experiments. YL and BF analyzed the data. YL and YY wrote the manuscript.

### Conflict of Interest

The authors declare that the research was conducted in the absence of any commercial or financial relationships that could be construed as a potential conflict of interest.
