# Supplementary material for: An Integrated Three-Long Non-coding RNA Signature Predicts Prognosis in Colorectal Cancer Patients
Source: Front Oncol. 2019 Nov 22;9:1269. doi: 10.3389/fonc.2019.01269 (PMC6883412; doi:10.3389/fonc.2019.01269)
Supplement: Supplementary file 1 [file Table_1.docx]

**An integrated three-long noncoding RNA signature predicts prognosis in colorectal cancer patients**

# Supplementary Figures and Tables

## Supplementary Figures


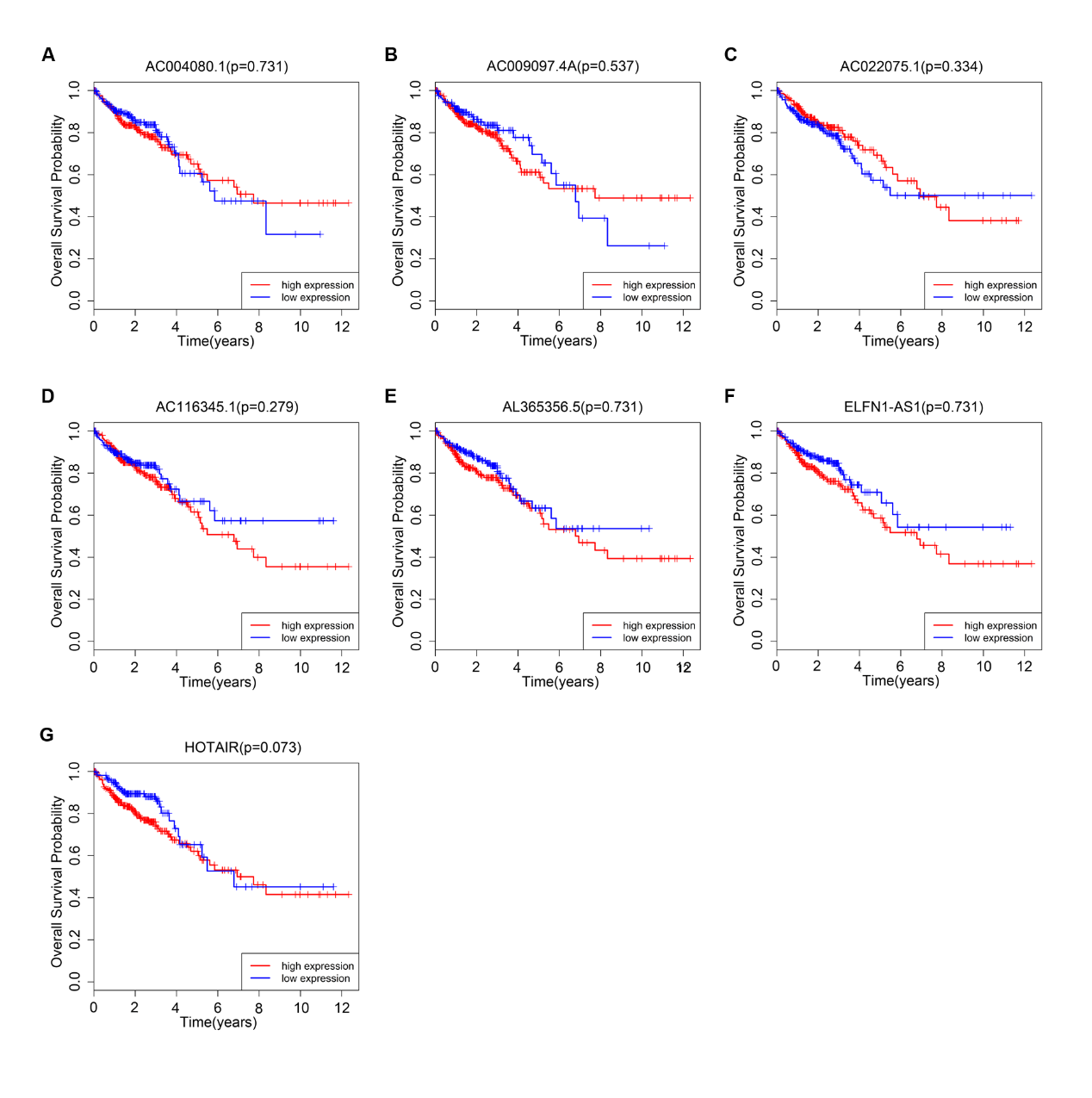


Supplementary Figure 1. The Kaplan-Meier curve of seven prognostic lncRNAs in CRC patients collected from TCGA cohort. The Kaplan-Meier curve for (A) AC004080.1, (B) AC009097.4A, (C) AC022075.1, (D) AC116345.1, (E) AL365356.5, (F) ELFN-AS1, (G) HOTAIR.


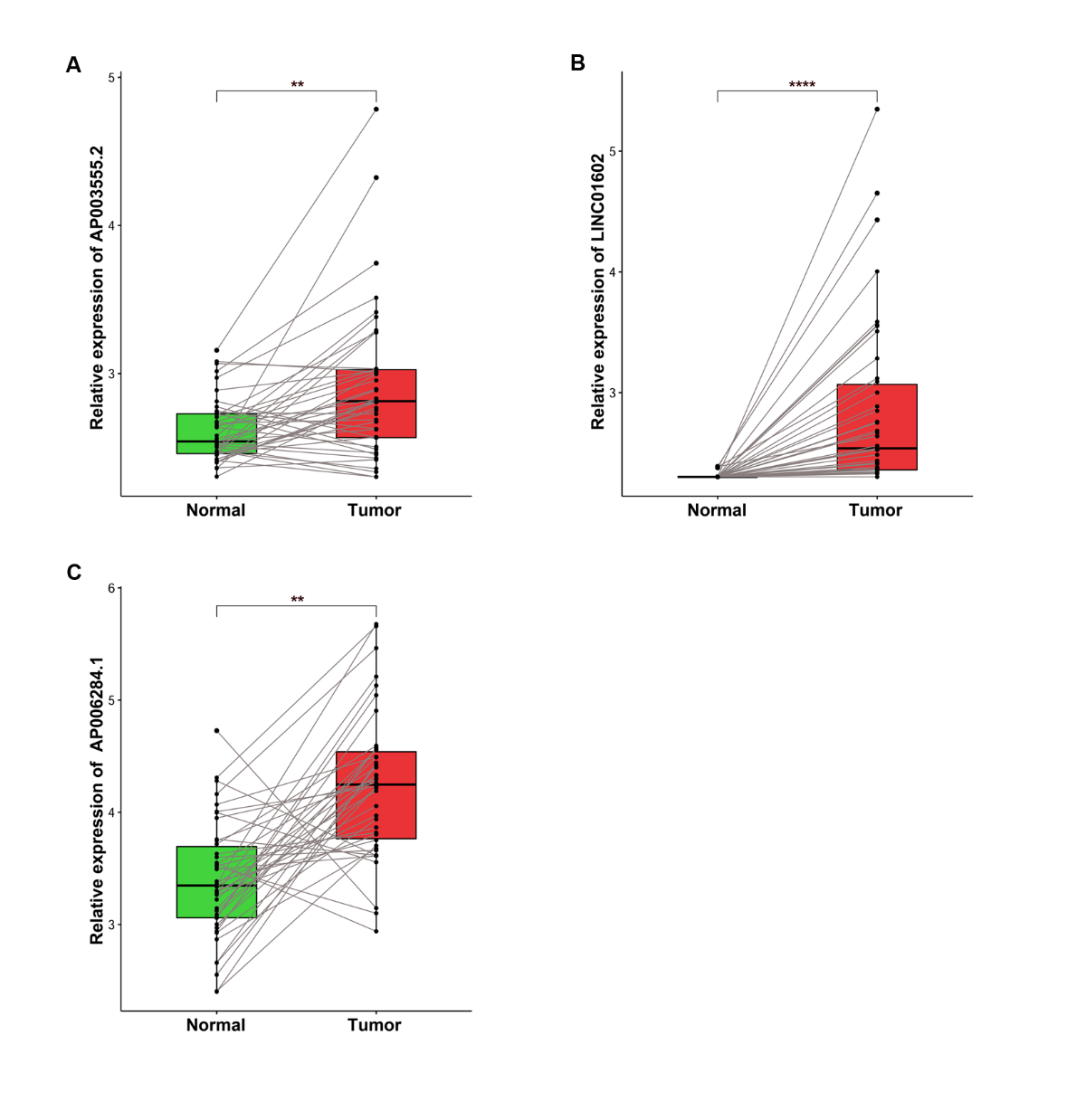


Supplementary Figure 2. The relative expression level of 3 lncRNAs (A) AP003555.2, (B) LINC01602, (C) AP006284.1 between 42 CRC tissues and their paired adjacent non-cancerous tissues.


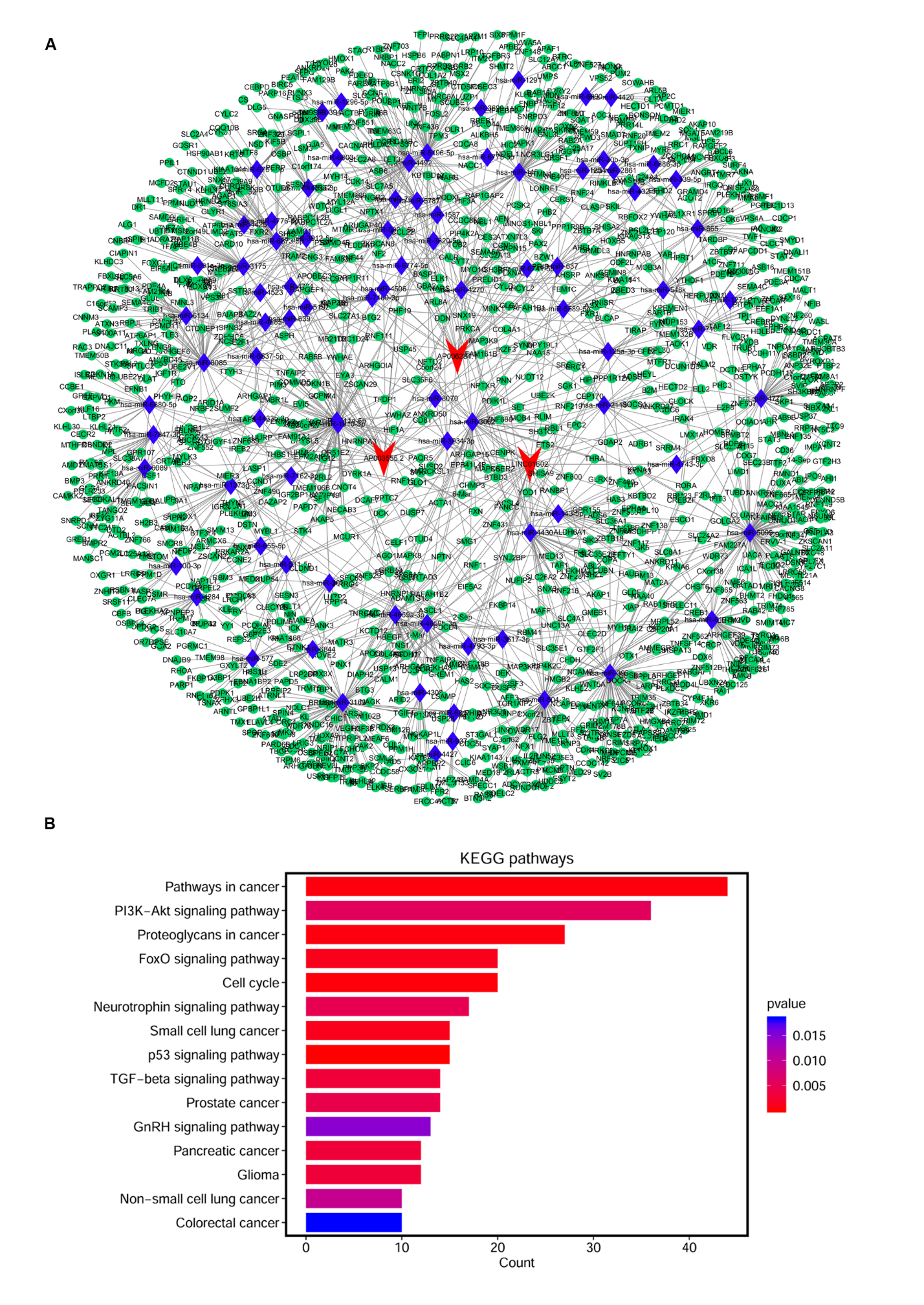


Supplementary Figure 3. The ceRNA network based on three prognostic lncRNAs and KEGG pathway analysis of selected mRNAs. Red nodes represent LncRNAs, blue nodes represent miRNAs and green nodes represent mRNAs (A); Top 15 pathways for selected mRNAs (B).

Supplementary tableSupplementary table 1. Three lncRNAs significantly correlated with prognosis of CRC.

| Gene name | Ensemble ID | | Chr. | | Coordinate | Primer sequence (5'->3') | |
| --- | --- | --- | --- | --- | --- | --- | --- |
|  |  |  |  |  |  | Forward primer | Reverse primer |
| AP003555.2 | | ENSG00000254605 | 11 | 70,014,858-70,021,059 | | GTCTGTAAAGGACGCACCCA | GGAGCCATTTTGGGCGTTTT |
| AP006284.1 | | ENSG00000254815 | 11 | 557,595-560,107 | | CAGTGACAGGCACTCACCTA | TCTCCTGGAGCAGAGGGAATA |
| LINC01602 | | ENSG00000205293 | 8 | 57,855,500-57,984,126 | | TAACACCAGGGTTGCTGTGAG | GCAGGGCTGCCATATAAAACC |
|  | |  |  |  | |  |  |
